# N6-methyladenosine modification positively regulate Japanese encephalitis virus replication

**DOI:** 10.1186/s12985-023-02275-w

**Published:** 2024-01-19

**Authors:** Min Yao, Zhirong Cheng, Xueyun Li, Yuexiang Li, Wei Ye, Hui Zhang, He Liu, Liang Zhang, Yingfeng Lei, Fanglin Zhang, Xin Lv

**Affiliations:** 1 Department of Microbiology, Airforce Medical University, Xi’an, 710032 Shaanxi China; 2 College of Life Science, Yan’an University, Yan’an, 716000 Shaanxi China; 3 College of Basic Medicine, Yan’an University, Yan’an, 716000 Shaanxi China

**Keywords:** *Flavivirida*, Japanese encephalitis virus, N6-Methyladenosine modification, MeRIP-seq, METTL3

## Abstract

N6-methyladenosine (m6A) is present in diverse viral RNA and plays important regulatory roles in virus replication and host antiviral innate immunity. However, the role of m6A in regulating JEV replication has not been investigated. Here, we show that the JEV genome contains m6A modification upon infection of mouse neuroblast cells (neuro2a). JEV infection results in a decrease in the expression of m6A writer METTL3 in mouse brain tissue. METTL3 knockdown by siRNA leads to a substantial decrease in JEV replication and the production of progeny viruses at 48 hpi. Mechanically, JEV triggered a considerable increase in the innate immune response of METTL3 knockdown neuro2a cells compared to the control cells. Our study has revealed the distinctive m6A signatures of both the virus and host in neuro2a cells infected with JEV, illustrating the positive role of m6A modification in JEV infection. Our study further enhances understanding of the role of m6A modification in *Flaviviridae* viruses.

**Supplementary Information:**

The online version contains supplementary material available at 10.1186/s12985-023-02275-w.

## Introduction

Vector-borne RNA viruses, such as Japanese encephalitis virus (JEV), tick-borne encephalitis virus (TBEV), West Nile virus (WNV), dengue virus (DENV), yellow fever virus (YFV) and Zika virus (ZIKV), known as *Flaviviruses*, can cause a variety of severe diseases including hepatitis, vascular shock syndrome, encephalitis, acute flaccid paralysis, congenital abnormalities and fetal death [23]. As a neurotropic virus, JEV is a leading cause of epidemic encephalitis in Asia and parts of the Western and South Pacific Ocean [7]. Approximately 68,000 cases of Japanese encephalitis (JE) are reported in Asia every year, posing a serious threat to public health and safety. JEV is a positive-sense, single-stranded, enveloped RNA virus, its genome comprising of a 5’ untranslated region (5’ UTR), an open reading frame (ORF), and a 3’ untranslated region (3’ UTR). The ORF encodes three structural proteins including capsid, membrane or precursor membrane (prM) and envelope (E) protein, and seven nonstructural proteins (NS1, NS2A, NS2B, NS3, NS4A, NS4B, and NS5) [2]. Among them, NS3 and NS5 proteins form a multienzyme complex which is responsible for both positive and negative viral RNA syntheses, as well as 5’ capping [31].

The m6A methylation is one of the most prevalent internal post-transcriptional modification of RNA in eukaryotes [4]. The m6A modification process is reversibly modulated by three parts: the writer, reader and eraser. The writer refers to m6A methyltransferase complex, which mainly consisting of methyltransferase-like 3 (METTL3) and METTL14 [18, 35]. METTL3 functions as the catalytic subunit, while METTL14 serves as a scaffold for RNA binding. After deposition, m6A can be dynamically removed by two demethylases, Fat mass and obesity-associated protein (FTO) and ALKB homolog 5 (ALKBH5), known as eraser [12, 45]. Moreover, the m6A is recognized by the YTH domain-containing proteins, which are referred to as readers and exert regulatory functions through selective recognition of methylated RNA. YTHDF1 and YTHDF2 facilitate the translation and degradation of target mRNAs, respectively, while YTHDF3 synergizes with these two proteins [27, 34, 36].

The addition of m6A onto mRNA, which occurs at the consensus motif DRA^m^CH (where D = G/A/U, R = G > A, and H = U/C/A), and plays an important regulatory role in an array of physiological and pathological processes [25, 43]. The role of m6A and its machinery in virus-host interactions has been investigated for years. Increasing evidence indicates that m6A modification is present in viral RNA to exert pro-viral or antiviral actions through regulating viral life cycle and host antiviral innate immunity [15, 21, 24]. It has been reported that m6A modification is present in the genomes of several *Flaviviridae* viruses, such as hepatitis C virus(HCV), ZIKV, DENV, YFV, and WNV [6, 9]. Knockdown of METTL3 and METTL14 resulted in a significant increase in HCV and ZIKV replication and virus titer, suggesting that m6A may have a negative effect on HCV and ZIKV infection [6, 16]. In addition, *Flaviviridae* infection can modify m6A modification of specific cellular mRNAs, including RIOK3 and CIRBP, and this, in turn, can influence replication of the DENV, ZIKV, and HCV [5]. However, the association between m6A and JEV and the mechanism by which m6A regulates JEV infection are still largely unknown.

Here, we found that the m6A modification sites located on the JEV genome undergo dynamic changes during the progression of infection. A decrease in JEV replication and progeny particle release is observed when METTL3 is knocked down in neuro2a cells. This reduction in JEV replication is attributed to METTL3 knockdown stimulating the innate immune response. Our study has revealed the unique viral and host m6A modifications profiles during JEV-infection, thereby demonstrating the role and regulatory mechanism of m6A modification in *Flaviviridaes* virus. This highlights the complexity and importance of m6A modification during viral infection.

## Material and method

### Ethics statement

All animal experiments were reviewed and approved by the Animal Care and Use Committee of the Laboratory Animal Center, Air Force Medical University. The number of Animal Experimental Ethical Inspection is 20,200,410. All experiments were carried out complying with the recommendations in the Guide for the Care and Use of Laboratory Animals.

### Cells and virus

The JEV P3 strain (GenBank accession No. U47032.1) was propagated in the brains of 3-day-old inbred C57BL/6 J suckling mice and titrated by conventional plaque assay. The neuroblast cell line neuro2a and baby hamster kidney (BHK) cells were cultured in Dulbecco’s modified Eagle’s medium (DMEM; Gibco, United States) containing 10% fetal bovine serum (FBS; Gibco, United States) and 1% penicillin–streptomycin combination.

### Virus infections

Neuro2a cells were seeded in six-well plate at a density of 2 × 10^5^ per well overnight. Then the cells were infected with JEV (MOI = 0.5). After incubation for 2 h, the virus suspension was removed, and fresh DMEM was added. The cells and supernatant were harvested at indicated time points for RT-PCR, western blot, and conventional plaque assay respectively.

### Animal infection experiment

6-week-old male C57BL/6 J mice (average weight 18 to 20 g) were purchased from Laboratory Animal Center of Air Force Medical University and maintained in individually ventilated animal cages in the specific pathogen-free environment. Mice were randomly divided into two groups, six mice per group. 1 × 10^6^ PFU in 20 µL DMEM of JEV P3 strain was injected into the foot pads of mice, while the same volume of sterile DMEM was injected into the control group. Samples of whole brain tissue were collected from JEV-infected mice displaying mild symptoms (e.g. ruffled fur and curling behavior), moderate symptoms (e.g. arched back, tremor, and lethargy), and severe symptoms (e.g. hind limb paralysis, frequent blinking, and orbital hemorrhaged) [7, 47] for RT-PCR and western blot analysis.

### Plaque-forming assay

BHK-21 cells (2 × 10^5^/well) were seeded into six-well culture plates and incubated overnight. Supernatants of cells were removed and then cells were washed twice with PBS. Serial tenfold diluted samples collected from neuro2a were added and incubated at 37 ℃ for 2 h. After viral adsorption, cell monolayer was covered with overlay medium (25 mL 4 × DMEM, 25 mL ddH_2_O, 50 mL 4% methyl cellulose and 2 mL FBS) for 5 days. Then, the overlay medium was removed, and the cell monolayer was stained with 1% crystal violet.

### siRNA transfection and overexpression-plasmids transfection

METTL3, METTL14 and WTAP, YTHDF1, YTHDF2, YTHDF3 specific siRNAs were designed and synthesized by GenePharma biotech company. Lipofectamine RNAiMAX reagent (13,778–150, Invitrogen, USA) was utilized to transfect siRNAs into neuro2a cells as instructed by the manufacturer. The potency of interference was evaluated through RT-PCR or western blot. GV657-METTL3 and GV657-YTHDF1 plasmids, designed by Shanghai GeneTechnology, are used to overexpress METTL3 and YTHDF1. Following 24 h of transfection of neuro2a cells with siRNA or plasmids, the cells were infected with JEV for an additional 48 h before being collected for RT-PCR or western blot analysis.

### RT-PCR

The mouse brains were homogenized using the TissueLyser (30 Hz, 10 min) (Qiagen, Hilden, Germany). Total RNA from mouse brains and neuro2a cells was extracted with E.Z.N.A. Total RNA Kit (R6834-02, Omega, USA) in accordance with the manufacturer’s instructions. The cDNA was prepared by reverse transcription with the total RNA as template using the Hifair II 1st strand cDNA synthesis superMix (Yeasen, China). RT-PCR experiments were performed using 2 × qPCR SmArt Mix (SYBR Green) (DY20301, DEEYEE, China). Quantitative PCR reactions were performed in triplicate and the mean Ct value of each sample was used for data analysis. The level of mRNA expression was normalized with β-actin, and the data are shown as the relative change to the corresponding reference for each group. The primers used in this research were provided in (Additional file 1: supplementary Table 1).

### Western blot

Brain tissue from a mouse or cells was extracted with a radioimmunoprecipitation assay (RIPA) buffer, which contained phenylmethanesulfonyl fluoride (PMSF) and phosphatase inhibitors, and the total protein was then quantified using a Protein Reagent Assay BCA Kit (23,227, Thermo, USA). Sixty micrograms of protein from each sample was loaded, electrophoresed using 12% SDS-PAGE gels, and then transferred onto PVDF membranes (Millipore, Billerica, MA, United States). The membranes were blocked with 3% BSA at room temperature for 60 min, after which they were incubated with the primary antibodies (listed in Additional file 1: supplementary Table 1) for an overnight period at 4 ℃. Then, the blots were incubated with the corresponding IRDye® 680/800-labeled secondary antibodies for 2 h at room temperature. An infrared imaging system was employed to visualize the blots (Odyssey, LI-COR, United States).

### Inhibitor treatments

The pan-Janus protein tyrosine kinase (JAK) inhibitor Ruxolitinib Phosphate (S5243, Selleck) was reconstituted in DMSO as a 5 mM stock solution. Neuro2a cells were transfected with siMETTL3 and 24 h later, they were infected with JEV (MOI = 0.5) for 2 h. Subsequently, the medium was replaced with a culture medium containing ruxolitinib (2 µM) and the cells were incubated for an additional 48 h. Relative JEV RNA levels in JEV-infected METTL3 knockdown and control cells treated with or without ruxolitinib were analyzed by RT-PCR.

#### Sample Collection for MeRIP Analysis

1 × 10^7^ neuro2a cells were seeded in T75 cell culture bottle until completely adherent. After mock or JEV-infected for 24 and 48 h, the cells were washed three times with PBS and scraped. Harvested cells were immediately snap-frozen in liquid nitrogen and stored at -80 ℃ for measurement. A comprehensive overview of the MeRIP-seq data processing and alignment, MeRIP-seq peak-calling, motif discovery and metagene analysis is provided in supplementary materials (listed in Additional file 2).

#### Statistical analysis

Statistical analysis was performed with the GraphPad Prism 8.0 (GraphPad, Inc, USA). Experiments were independently repeated the indicated number of times listed in the figure legend. Representative data was exhibited as the means ± SEM. Quantitative data was compared using Student’s t test. In addition, correlational analysis of gene expression was conducted with linear regression. *P*-values for every result were labeled on figures, and *p* < 0.05 was considered statistically significant (*p < 0.05, **p < 0.01, ***p < 0.001, ****p < 0.0001, ns, non-significant).

## Results

### m6A modification is present in the JEV genome

**Fig. 1:**
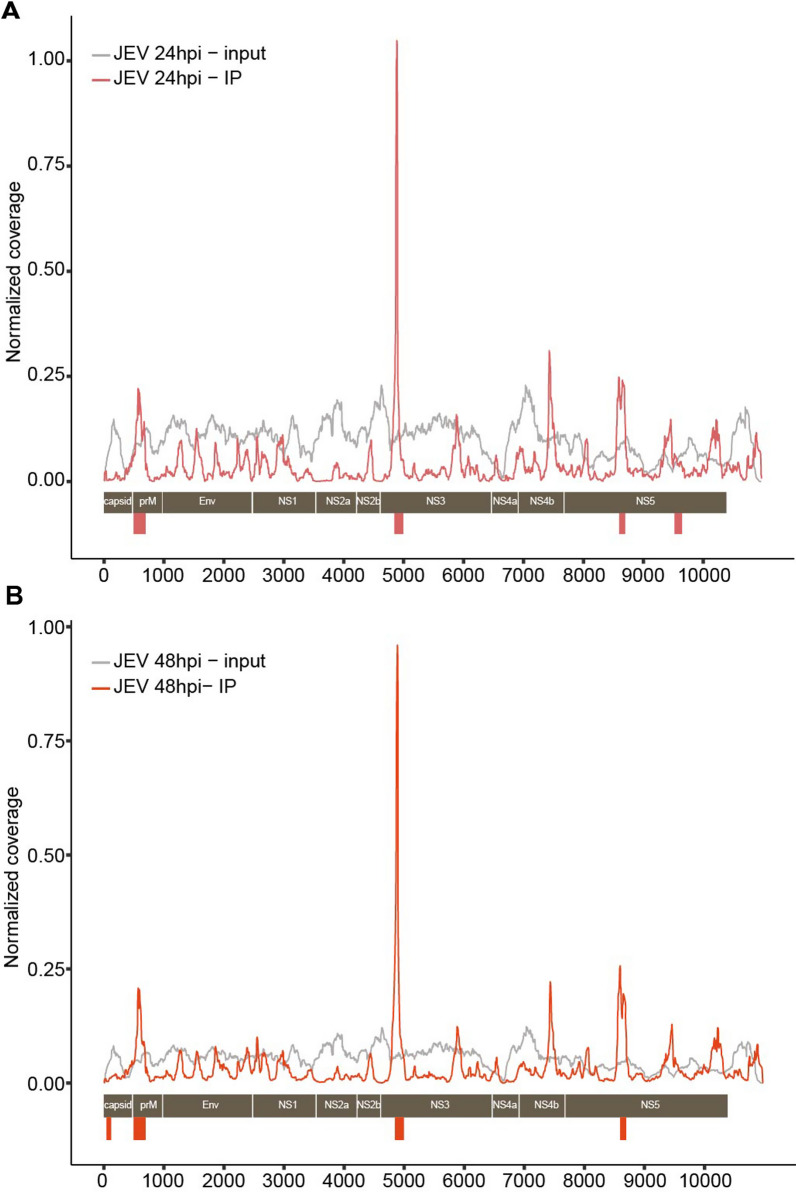
JEV RNA Is modified by m6A. **A** MeRIP-seq of RNA isolated from JEV-infected neuro2a cells at 24hpi and 48hpi has revealed a map of m6A in the JEV genome, with the signal from IP samples indicated by a red line and the baseline signal from input samples shown as a gray line. A schematic of the JEV genome is presented to illustrate the location of the m6A-enriched sequences (Red bars) (n = 1; FDR-corrected q value < 0.01)

In order to determine whether the JEV genome contains m6A modifications, we extracted RNA from mock- or JEV-infected neuro2a cells at at 24 hpi and 48 hpi, respectively, and subjected it to m6A-specific antibody immunoprecipitation followed by high-throughput sequencing (m6A-seq). We used a stringent peak calling method (false discovery rate[FDR] < 0.01) and sequenced the methylome of JEV with very high depth, leading to the identification of statistically significant peaks. The p-values for all identified peaks were < 1e-5. Total five discrete m6A peaks were identified spanning the full length of the JEV genome, located in the regions of the 5’ UTR/capsid, prM, NS3, and NS5, respectively (Table 1). A m6A peak was detected in the prM (495–695nt), NS3 (4845–4995nt), and NS5 (8595–8695nt) regions at both 24 hpi and 48 hpi. While the m6A peak located in NS5 (9520-9645nt) was only detected at 24 hpi and m6A peak located in 5’ UTR/capsid appeared only at 48 hpi. It indicated that some m6A modification sites appear to remain conserved during JEV infection, whereas others undergo dynamic changes. Remarkably, the m6A peak located in NS3 (4845–4995nt) showed the highest enrichment at both 24 hpi and 48 hpi, as illustrated in Fig. 1 and Table 1. The presence of two m6A modification sites is observed only in the NS5 region. Due to the RNA helicase activities of NS3 and the RNA-dependent RNA polymerase activity of NS5, it is likely that the m6A modification sites on the NS3 and NS5 regions are of great significance in the replication of JEV.

**Table 1:** Nucleotide location and score of the five m6A peaks identified in JEV RNA by MeRIP-seq

m6A Peak Number	Start	End	Score -log2(adj.pvalue)	Region of JEV genome	Timepoint
1	45	120	0.15	5'UTR/caspid	48 hpi
2	495	695	1.62	prM	24 hpi/48 hpi
3	4845	4995	2.98	NS3	24 hpi/48 hpi
4	8595	8695	0.11	NS5	24 hpi/48 hpi
5	9520	9645	0.13	NS5	24 hpi

**Fig. 2:**
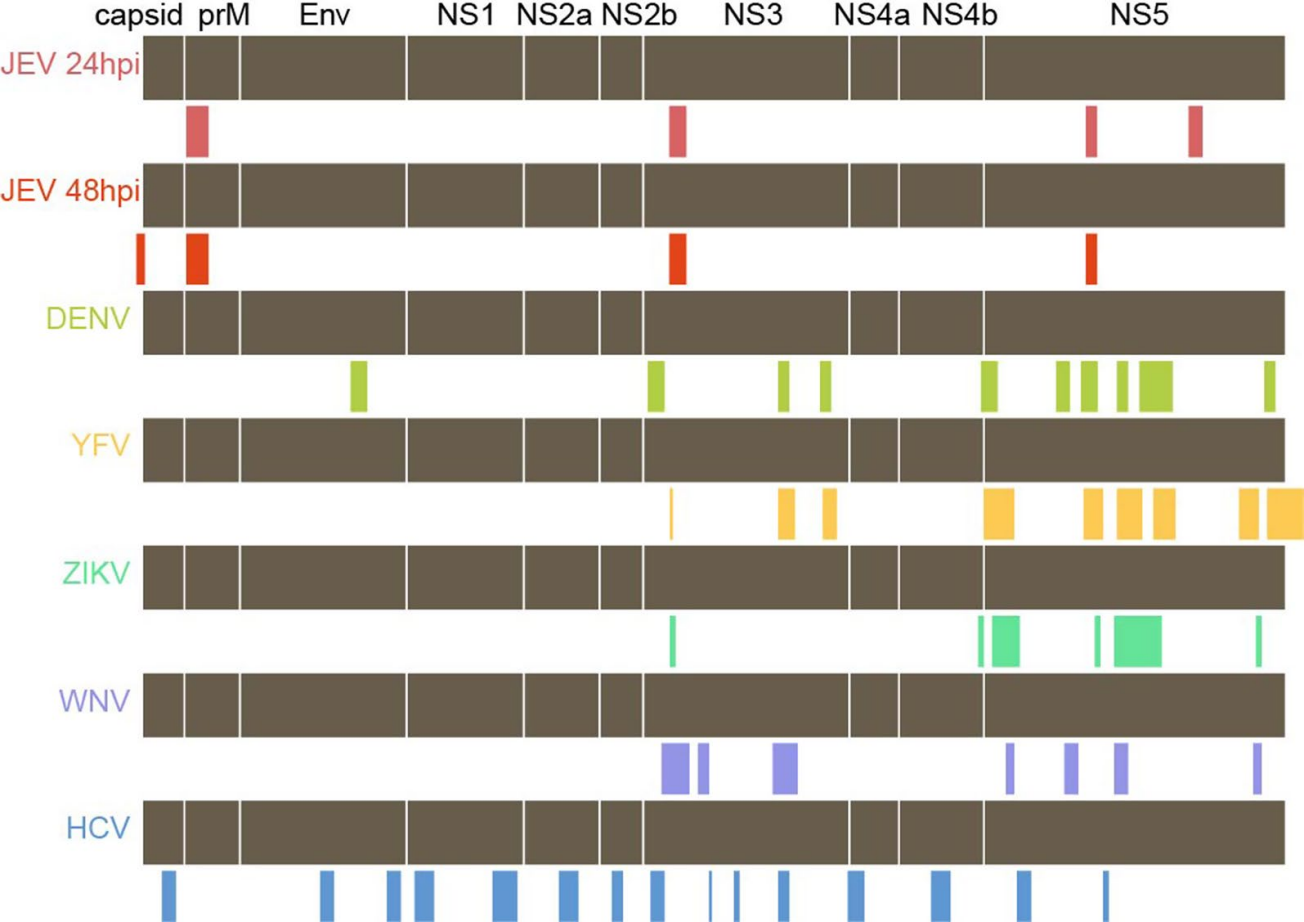
Alignment of m6A sites in the genomes of six *Flaviviridae* viruses, including JEV, DENV, YFV, ZIKV, WNV and HCV. Data about the m6A modification sites on the genomes of DENV, YFV, ZIKV, WNV, and HCV has been obtained from [6]'s study

It has been reported that m6A is present in the genomes of several *Flaviviridae* viruses. To evaluate whether the m6A sites on the genome of *Flaviviridae* viruses is conserved, we compared the m6A sites on the JEV genome to those of five other *Flaviviridae* viruses. Our investigation uncovered that m6A modifications are more frequent in the NS3 and NS5 regions of the six *Flaviviridae* viruses (Fig. 2). These sites, located on NS3 and NS5, span the regions of 4845-4995nt and 8595-8695nt respectively. A distinctive m6A site is present in the prM (495-695nt) region of the JEV genome, a feature that is not observed in other *Flaviviridae* viral genomes. The prM protein is largely responsible for the assembly of viruses and their virulence, suggesting that m6A modification of prM may be involved in this process [29, 39, 41].

The findings collectively demonstrated that the JEV genome undergoes m6A modification during its infection of neuro2a cells. The genome of *Flaviviridae* viruses contains both conventional and specific m6A sites. Moreover, m6A sites on the JEV genome undergo dynamic changes during the infection of neuro2a cells.

### JEV infection caused the host cells to gain more m6A peaks

**Fig. 3:**
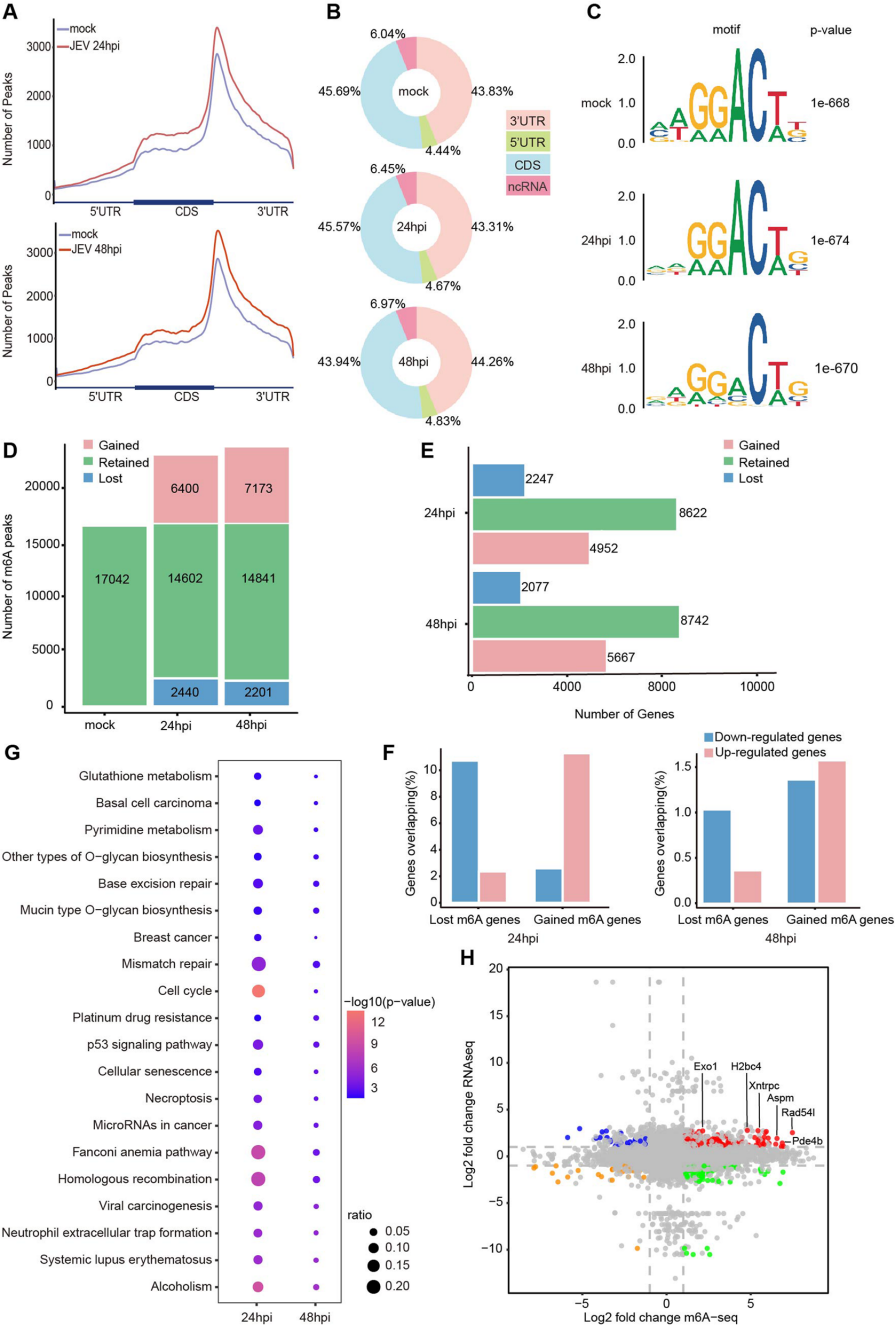
JEV infection influences the m6A methylome of host cell. **A** Analysis of m6A peak enrichment in mock (gray line) and JEV-infected neuro2a cells (red line) via metagene analysis reveals annotation of 5' UTR, 3' UTR, and CDS at 24 hpi and 48 hpi, respectively. **B** A pie chart illustrates the relative proportions of total m6A peaks in the 5' UTR, CDS, 3' UTR and ncRNA of host cell RNA transcripts. **C** The top consensus sequences for m6A methylation in mock and JEV-infected neuro2a cells at 24 hpi and 48 hpi. **D** The total number of m6A peaks that were classified as gained, retained, or lost after 24-h or 48-h infection with JEV in comparison to neuro2a cells was determined. **E** Bar plots are used to depict the quantity of genes that have gained, retained, or lost m6A in neuro2a cells after 24 hpi and 48 hpi. **F** A bar graph illustrating the percentages of up- or down-regulated genes that overlap with either gained or lost m6A genes. **G** KEGG analysis of genes which were up-regulated and gained m6A peak upon JEV infection at 24 hpi and 48 hpi revealed the top 20 enriched pathways. The size of the dots indicates the gene enrichment ratio, while their color reflects the log10 level of significance. **H** A nine-quadrant graph was generated to evaluate the correlation between transcriptome and m6A modification between mock and JEV-infected neuro2a cells. The abscissa of the graph is the fold change of the m6A peak (taken log2) while the ordinate is the fold change of the transcriptome (taken log2). The red dots signify genes that are both up-regulated in the transcriptome and m6A peaks, the orange dots represent genes that are down-regulated in both the transcriptome and m6A peaks, the blue dots identify genes that are up-regulated in the transcriptome but down-regulated in m6A peaks, and the green dots indicate genes that are down-regulated in the transcriptome but up-regulated in m6A peaks. Meanwhile, the gray dots signify genes and m6A peaks that are not differentially expressed. DiffBind was employed to assess the rate of RNA methylation while DESeq2 was utilized to analyze the differential expression of RNAs between different groups. Genes/peaks with a false discovery rate (FDR) below 0.05 and an absolute fold change ≥ 2 were considered to be significantly differential. The most differentially expressed genes were also labeled

Since viral infection impacts the m6A methylome of the host cells [19], we investigated if and how JEV infection would affect the m6A methylome of the neuro2a cells. ExomePeak2, a strict peak-calling tool, identified 17,042, 21,002, and 22,014 m6A peaks from 9312, 10,199, and 10,676 m6A-modified genes, respectively, in mock and JEV-infected neuro2a cells at 24 hpi and 48 hpi (Additional files 3, 4, 5, 6, 7). Results of the global distribution of m6A peak density indicated that JEV infection elevated m6A levels in the coding sequence and 3’ UTR regions of the transcriptome at both 24 hpi and 48 hpi. (Fig. 3A). JEV infection did not alter the distribution of global m6A peak on any region of mRNA (Fig. 3B). The m6A peaks from mock and JEV-infected neuro2a cells were found to be highly enriched with the "GGAC" sequence, which is in agreement with the DRACH motif (Fig. 3C). Following 24 h of JEV infection, the host cells showed exhibited 6400 gained peaks and 2440 lost peaks. After 48 h, the host cells experienced a further gain of 7173 m6A peaks and a loss of 2201 m6A peaks (Fig. 3D). Our analysis showed that JEV infection caused the host cells to gain more m6A peaks, as evidenced by the increased number of m6A genes at 24 hpi and 48 hpi (Fig. 3E); thus, indicating that JEV infection has a significant influence on the m6A modification of the host cells. The transcripts that display a significant difference in m6A abundance between infected and non-infected cells are listed in the (Additional files 3, 4, 5, 6, 7) (*P* ≦0.05; fold change ≧1.5).

To gain further insight, we studied the relationship between epitranscriptome and the transcriptome of the host cells in mock and JEV-infected neuro2a cells at 24 hpi and 48 hpi. The results revealed that the majority of genes with gained m6A peaks experienced an increase in expression levels, whereas those with lost m6A peaks exhibited largely decreased expression levels (Fig. 3F). Subsequently, the up- and down-regulated genes with gained m6A peak at 24 hpi and 48 hpi were identified for Kyoto Encyclopedia of Genes and Genomes (KEGG) analysis (Fig. 3G). Our analysis revealed that genes which were up-regulated and had gained m6A peak were mainly associated with the Fanconi Anemia pathway, Homologous Recombination, Cell Cycle, Mismatch Repair, Glutathione Metabolism, and Viral Carcinogenesis (Fig. 3G). Exo1, H2bc4, Xntrpc, Rad54l, Aspm, and Pde4b (red dot) were amongst the genes that had increased levels of m6A modification and were significantly upregulated in infected cells. Our results demonstrate that following infection with JEV, the m6A modification of host cell transcripts is increased, resulting in a modification of host gene expression.

### JEV infection decreases the abundance of METTL3 in vivo

**Fig. 4:**
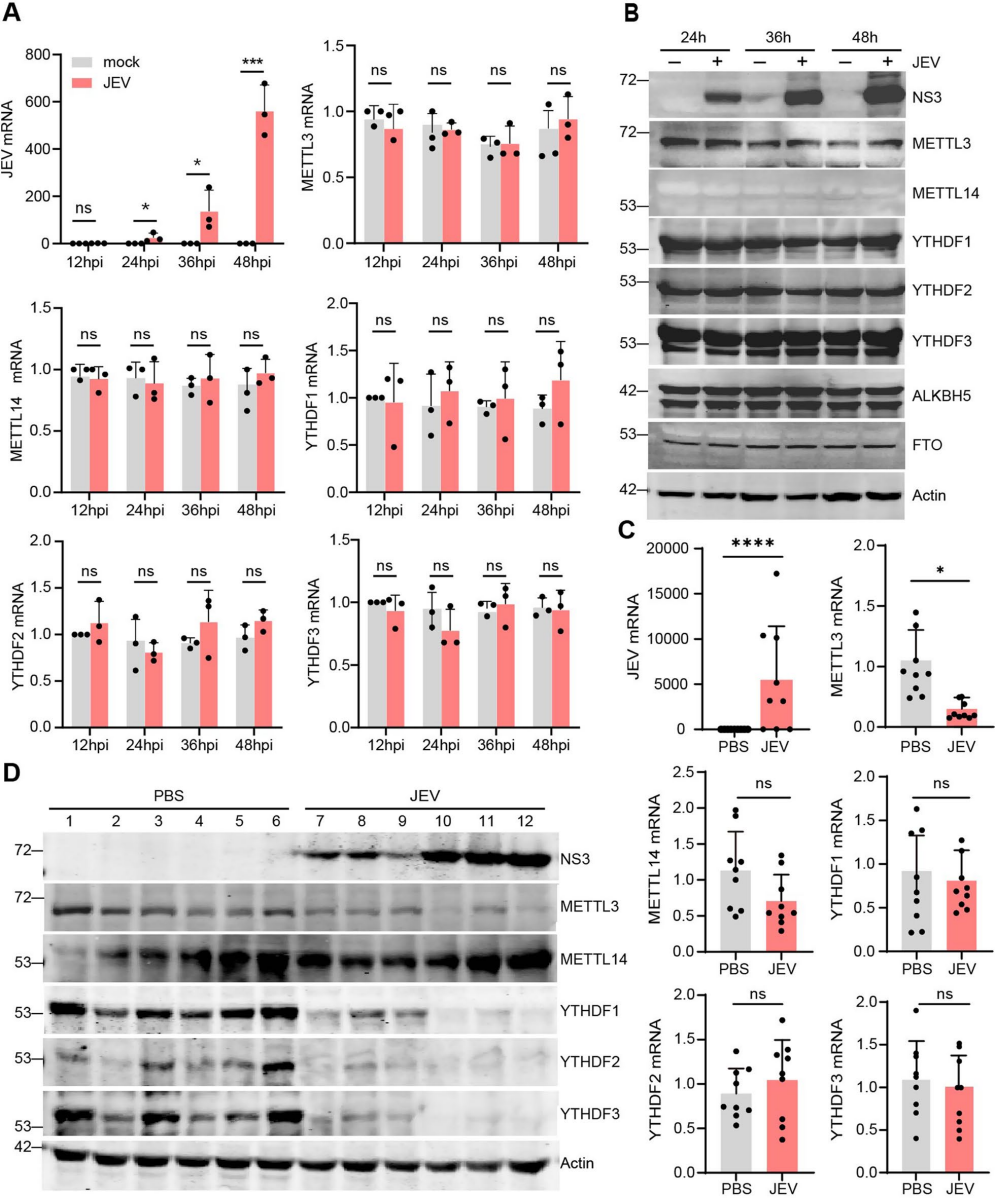
JEV infection decreases the expression of METTL3 in vivo. **A** Neuro2a cells were infected with JEV(MOI = 0.5) at the indicated time, and the levels of m6A writers and readers in the RNA were subsequently assessed using RT-PCR. **B** The expression of m6A writers and readers was analyzed by immunoblotting with the indicated antibodies, and β-actin was used as a loading control. **C** C57BL/6 J mice aged 4—6 weeks were given a footpad injection of either JEV or PBS, and their brains were harvested at mild, moderate, and severe stages of the onset (n = 6).The mRNA level of m6A writers and readers was evaluated by RT-PCR. The data represent the change relative to the level of the PBS group. Data are shown as the mean ± SD. Three independent experiments were performed. Statistical significance was determined by Student’ s t-test. *p < 0.05. **D** The expression of m6A writers and readers was evaluated by immunoblotting with the indicated antibodies. β-actin was used as a loading control

To further explore the effects of JEV infection, we examined whether it had an influence on the expression of m6A writers, readers, and erasers both in vitro and in vivo. Evaluation of mRNA levels and protein expression of m6A writers, readers and erasers was conducted in neuro2a cells following JEV infection. RT-PCR analysis revealed that mRNA levels of METTL3, METTL14, YTHDF1, YTHDF2, and YTHDF3 were not significantly altered at 24 hpi and 48 hpi (Fig. 4A). Similarly, no changes in the expression of m6A writers, readers, and erasers were observed in response to viral NS3 expression after infection (Fig. 4B).

To further validate the expression of m6A writers and readers after JEV infection in vivo, brain tissues of mock- or infected-C57BL/6 J mice at mild, moderate, and severe stages of onset were harvested. Our findings indicated that the more severe the disease in mice, the higher the expression of NS3 protein in brain tissue (Fig. 4C and D). METTL3 mRNA level was significantly reduced in the brain tissue of JEV infected mice compared to the PBS group, however, METTL14, YTHDF1, YTHDF2, and YTHDF3 remained unchanged (Fig. 4C). Analysis of JEV-infected mouse brain tissue revealed a significant decrease in the expression of METTL3, YTHDF1, YTHDF2, and YTHDF3, while the expression of METTL14 remained unchanged (Fig. 4D). The findings revealed that JEV infection decreases the abundance of METTL3 in vivo.

### m6A modification positively regulates the replication of JEV

**Fig. 5:**
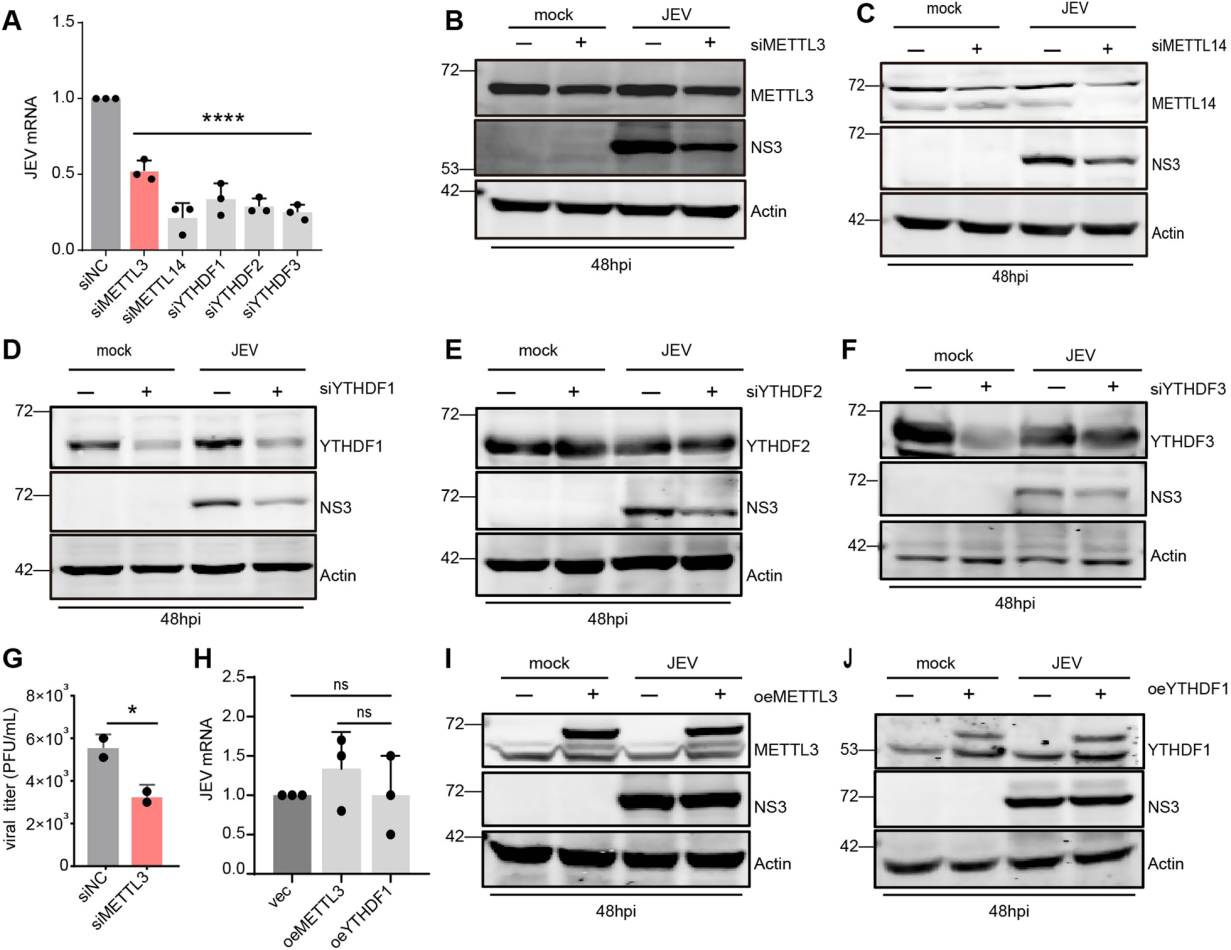
m6A modification positively regulates the replication of JEV. **A** RT-PCR analysis was conducted on neuro2a cells which had been transfected with a specific siRNA and infected with JEV (MOI = 0.5) for 48 h, with three replicates, to measure the relative JEV RNA level. All data are the means ± SD of the indicated number of replicates. Statistical significance of the difference was determined by Student’s unpaired t-test. **** *p* < 0.0001. **B-F** Immunoblot analysis was conducted to measure NS3 expression in neuro2a cells that had been transfected with the indicated siRNA and infected with JEV (MOI = 0.5) for 48 h. **G** The PFU assay was performed twice to determine the titers of JEV in the supernatants of siMETTL3-treated samples at 48hpi. **H-J** RT-PCR and Immunoblot analyses were conducted to assess NS3 expression in neuro2a cells that had been overexpressing either METTL3 or YTHDF1, and then infected with JEV( MOI = 0.5) for 48 h

Recent research has uncovered that the consequences of m6A methylation on viruses can differ, contingent on the virus, cell, or stage of infection. Therefore, we investigated how m6A modifications regulate the lifecycle of JEV in neuro2a cells. Our research revealed that the individual silencing of METTL3, METTL14, YTHDF1, YTHDF2 and YTHDF3 had no effect on JEV RNA level and NS3 expression at 24 hpi (Additional file 8: Supplementary Fig. 1A-F). However, at 48 hpi, a significant decrease in JEV replication was observed when these genes were silenced by siRNA (Fig. 5A-E). A PFU assay found that at 48 hpi, there was a consistent and considerable decrease in the production of JEV progeny viruses in METTL3 knockdown cells (Fig. 5G). We discovered that there was no considerable impact on JEV RNA levels or NS3 expression when METTL3 or YTHDF1 were overexpressed (Fig. 5H-J). Taken together, these results showed that the suppression of m6A writers and readers led to a reduction in JEV replication and protein expression, especially during the later stages of JEV infection in neuro2a cells, suggesting that m6A modification has a positive effect on JEV replication.

### METTL3 knockdown inhibits JEV replication by enhancing the innate immune response

**Fig. 6:**
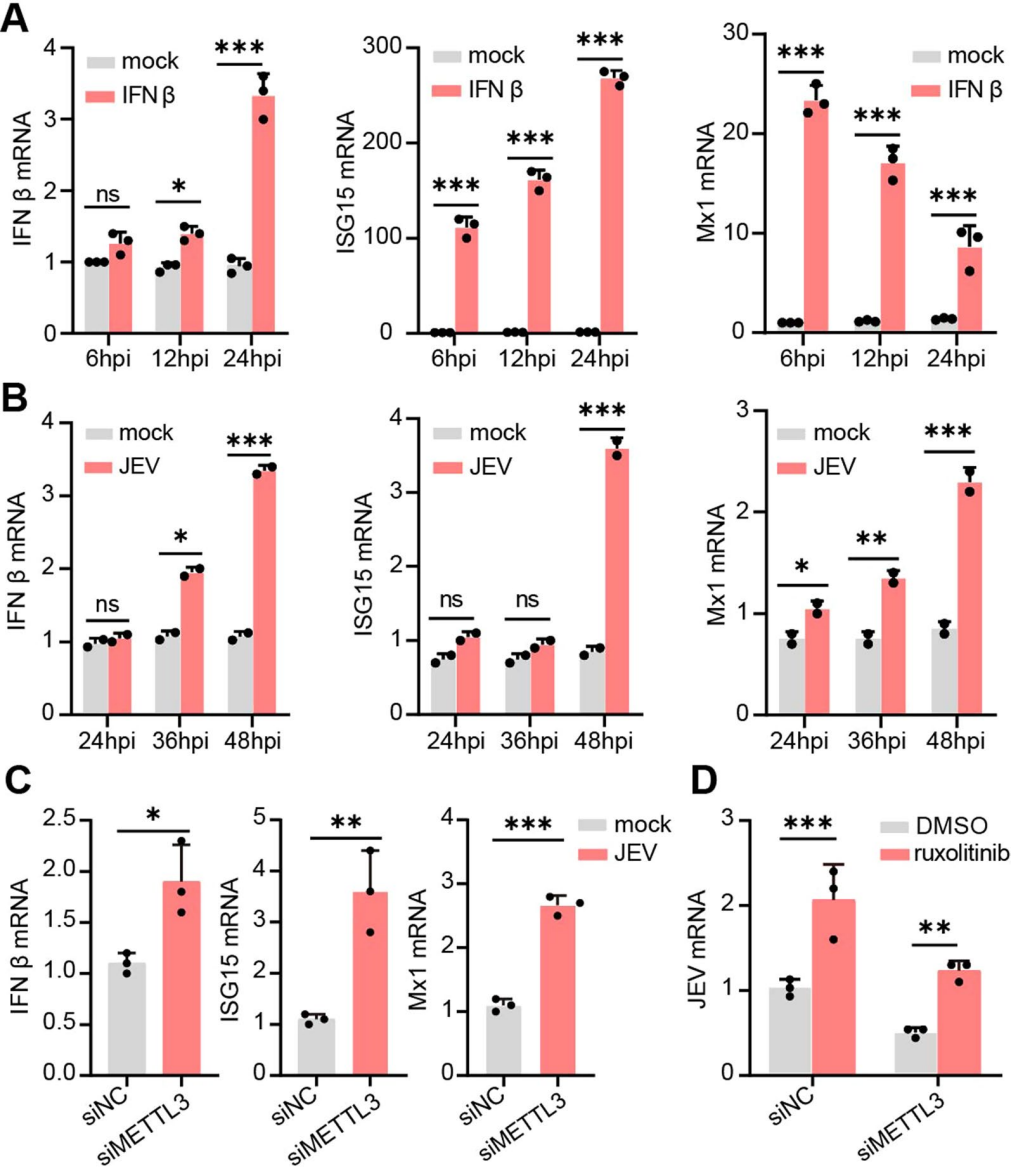
METTL3 knockdown inhibits JEV replication by enhancing the innate immune response. **A** Human interferon β was applied to neuro2a cells and the mRNA levels of IFN β, Mx1, and ISG15 were monitored at the selected time points. **B** Neuro2a cells were infected with JEV (MOI = 0.5), and the levels of IFN β, Mx1, and ISG15 mRNA were measured at the designated time points following the infection. **C** At 48 hpi, RT-PCR was conducted to measure the mRNA levels of IFN β, Mx1, and ISG15 in the METTL3 knockdown and control cells. **D** RT-PCR analysis was conducted to measure the relative JEV RNA level in JEV-infected METTL3 knockdown and control cells treated with or without ruxolitinib, at 48 hpi (n = 3). All data are the means ± SD of the indicated number of replicates. Statistical significance of the difference was determined by Student’ s unpaired t-test. *p < 0.05; **p < 0.01, *** *p* < 0.001; ns, nonsignificant

Research has demonstrated that when either METTL3 or METTL14 is depleted, the amount of type I interferon (IFN) and antiviral interferon-stimulated gene (ISG) expression increases in virus-infected cells [24, 26, 37]. Therefore, we examined if METTL3 depletion could impede JEV replication due to its potential to bolster innate immunity. After 12 and 24 h of human interferon β treatment, a significant increase in the levels of IFN β, ISG15, and Mx1 was detected (Fig. 6A). However, IFN β, ISG15, and Mx1 did not display an immediate increase after JEV infection at 12 and 24 hpi, instead increasing gradually and peaking at 48 hpi (Fig. 6B). Previous research has indicated that a delayed IFN β induction in SK-N-SH cells impairs the IFN response during early infection, which is corroborated by the observation that the IFN response is functional but delayed in neuro2a cells [30].

Further investigation revealed no significant differences in the transcript levels of IFN β, ISG15, and Mx1 between METTL3 knockdown cells and control cells at 24 hpi and 36 hpi (Additional file 9: Supplementary Fig. 2). Nevertheless, at 48 hpi, the transcript levels of IFN β, ISG15 and Mx1 were higher in METTL3 knockdown cells infected with JEV (Fig. 6C). Our findings indicate that METTL3 knockdown increases interferon response, leading to a decrease in JEV replication. To validate that this METTL3 knockdown-mediated inhibition of JEV replication is caused by an upregulation of interferon response, we implemented ruxolitinib to impede interferon signaling and ascertain if it would restore virus replication. Ruxolitinib is a highly potent and selective Janus kinase (JAK) 1 and 2 inhibitor that blocks the signaling downstream of the type I interferon receptors [17]. Inhibition of interferon signaling through ruxolitinib treatment was able to recover JEV replication in METTL3 knockdown cells (Fig. 6D). Data from the experiment demonstrates that the suppression of JEV in METTL3 knockdown cells is mainly due to a heightened interferon response.

## Discussion

m6A modifications have been found to have a range of effects on the life cycles of different viruses, such as impacting RNA stability, decay, transport, and protein translation. Despite this, the distribution, function, and regulatory mechanism of m6A modification in the JEV genome remains unknown.

Our MeRIP-seq analysis demonstrated that the JEV genome contains five m6A sites located in the 5’ UTR/capsid, prM, NS3, and NS5 regions. To confirm the m6A modification sites in the JEV genome, miCLIP assay, modified m6A individual-nucleotide-resolution cross-linking, and MeRIP-qRT-PCR should be conducted in future studies. Results have indicated that the m6A sites in the genomes of JEV and five other *Flaviviridae* viruses are both conserved and specific. It is noteworthy that the conserved m6A sites are mainly located in the NS3 and NS5 regions of all *Flaviviridae* viruses, which possess RNA helicases and RNA-dependent RNA polymerase activities essential for virus replication. Remarkably, the prM gene of JEV is the only one among the six *Flaviviridae* viruses to possess m6A modification. This prM serves as a chaperon for the folding of E protein and has been used as a vaccine component due to its immunogenicity-enhancing properties [3, 8]. Moreover, the 139 and 146 amino acids of ZIKV prM are associated with virus replication and pathogenicity in mice [11, 42]. Additionally, Flavivirus prMs have shown to significantly suppress interferon-I production by interacting with MDA5 and/or MAVS, suggesting that m6A modification of prM may be involved in host innate immune evasion [28].

Analysis of m6A sites in the JEV genome revealed dynamic alterations at 24 hpi and 48 hpi. Similarly, EBV transcripts displayed differential m6A modification at different infection stages among human nasopharyngeal carcinoma cells (NPC) biopsies, patient-derived xenograft tissues and cells [38]. Additionally, four and nine additional confident m6A peaks were identified at the SARS-CoV-2 genome at 24 hpi and 56 hpi respectively [19], and when it infects distinct cell lines, both conserved and different m6A sites were observed [44]. Furthermore, variations in m6A sites were observed among different ZIKV strains [16, 20]. Results suggested that the m6A sites of the virus could differ depending on the stage of infection, the cell line, and the viral strain, which could potentially affect the virus replication process. Regarding HCV, m6A sites located in the 3’ UTR of the HCV genome have been found to regulate RIG-I sensing activity [14], while m6A sites within the E1 region are associated with virion maturation [16] and those in the IRES region regulate cap-independent IRES-mediated translation [13]. Additionally, m6A modification of the 5’ ɛ stem-loop of HBV RNA has been observed to facilitate reverse-transcription activity, and m6A modification of the 3’ ɛ stem-loop reduces the viral RNA stability [10]. It is necessary to conduct further investigation in order to comprehend the role of each m6A site in the JEV genome and how these dynamic changes in JEV m6A modification in N2a cells affect virus replication or pathogenesis more thoroughly.

We found that JEV infection increases the number of m6A peaks in cellular RNAs, which could be a universal response to virus-induced stress similar to the m6A deposition changes noted in ZIKV and SARS-CoV-2 infection [16, 19]. Gokhale et al. discovered that infection by DENV, ZIKV, WNV, and HCV caused changes to the m6A modification of certain cellular transcripts [5], and that the activation of innate immunity and ER stress responses due to infection had a role in the different m6A modifications and in the alteration of translation or splicing of these transcripts [32]. The research suggests that m6A may act as a “messenger” between the virus and the host [46]. During viral infection, host cells modify the virus's genome through m6A modification, thus altering its pathogenicity,while the virus infection causes a shift in the m6A state of the host cells, as well as changes in the host gene expression, both of which could create a more favorable environment for the virus to replicate. Subsequently, we will delve deeper into this issue to evaluate the repercussions of JEV infection on the m6A modification state of host genes, as well as how host genes with modified m6A levels influence JEV replication.

No notable fluctuation in the expression levels of m6A writers, readers and erasers was observed in response to JEV infection in neuro2a cells, in agreement with the results obtained from MRC-5 cells infected with HCoV-OC43 [1]. In mouse brain tissue infected with JEV, a considerable decrease in the expression of METTL3, YTHDF1, YTHDF2, and YTHDF3 was observed. This is likely due to the fact that the mouse brain tissue consists of various kinds of cells, each of which responds differently to JEV infection. To gain a better understanding, it is essential to identify the primary cell type which is responsible for the reduction of M3 expression after JEV infection.

The presence of abundant m6A modification is essential for the life cycles of several viruses, as it is involved in both pro-viral and antiviral processes. Our investigations have demonstrated that the inhibition of JEV replication is observed upon the knockdown of METTL3, yet the deletion of METTL3 has been reported to promote HCV and ZIKV replication. It is likely that the regulatory mechanism of m6A modification on JEV is distinct from that of HCV and ZIKV, despite them belonging to the same family of *Flaviviridae*. We posit that this divergent regulatory result may be the consequence of distinctions in m6A sites on JEV and ZIKV genomes, as even on HCV genomes, different m6A sites have varying regulatory effects and can even produce opposite regulatory results.

Subsequent studies have uncovered that the m6A modification present in diverse locations of the HCV genome has distinct mechanisms and even opposite effects in regulating HCV replication. Consequently, previous research has demonstrated that m6A-deficient viral RNA induces a higher level of type I IFN, suggesting that m6A modification functions as a molecular indicator for innate recognition of self and non-self RNA [21, 22, 40]. Winkler *et a*l. reported that IFN β mRNA was modified by m6A and was significantly stabilized following depletion of METTL3, which inhibited HCMV growth [37]. Additionally, mice lacking METTL3 in small bowel intestinal epithelial cells (IECs) were found to be resistant to rotavirus (RV) infection due to increased Irf7 mRNA stability and increased expression of type I and III IFN [33]. Research has revealed that m6A modification plays a critical role in the innate immune response to viral infection. The results of MeRIP-seq reveal that IFN β in neuro2a cells is not modified by m6A, signifying that the knockdown of METTL3 does not affect the stability or degradation of IFN β (Additional files 3, 4, 5, 6, 7). We infer that the inhibition of JEV replication in neuro2a cells upon METTL3 knockdown, observed at 48 hpi rather than 24 hpi, is not due to variations in host recognition, which occurs in the early stages post-infection. Our findings suggest that the interferon and ISG mRNA levels are significantly increased in JEV-infected METTL3 knockdown cells, and the inhibition of JEV replication caused by METTL3 knockdown can be reversed by ruxolitinib treatment. This indicates that the enhanced type I IFN signaling is responsible for the inhibition of JEV by METTL3 knockdown.

In summary, we have shown that the host RNA methyltransferase system functions as a positive post-transcriptional regulator of JEV, by suppressing the innate immune response. Therefore, in our future research, we will explore other potential mechanisms that could explain the positive effect of m6A modification on JEV replication. Uncovering the specific mechanisms involved in the augmentation of JEV replication through m6A modification offers possibilities for the development of precision-targeted antiviral medications meant to interfere with these processes.

## Conclusions

Our current research has demonstrated that m6A modification is present in the JEV genome and is involved in the regulation of JEV replication. m6A modification positively regulates the replication of JEV. Our study further enhances understanding of the role and regulatory mechanism of m6A modification in *Flaviviridae* viruses.

### Supplementary Information

**Additional file 1.** A list of antibodies, siRNA and primers used in this study.

**Additional file 2.** A comprehensive overview of the MeRIP-seq data processing and alignment, MeRIP-seq peak-calling, motif discovery and metagene analysis is provided.

**Additional file 3.** A list of gained m6A peaks and m6A-modified genes in JEV-infected neuro2a cells at 24 hpi.

**Additional file 4.** A list of lost m6A peaks and m6A-modified genes in JEV-infected neuro2a cells at 24 hpi.

**Additional file 5.** A list of retained m6A peaks and m6A-modified genes in JEV-infected neuro2a cells at 24 hpi.

**Additional file 6**. A list of gained m6A peaks and m6A-modified genes in JEV-infected neuro2a cells at 48 hpi.

**Additional file 7**. A list of lost m6A peaks and m6A-modified genes in JEV-infected neuro2a cells at 48 hpi.

**Additional file 8**. RT-PCR and Immunoblot analysis was conducted on neuro2a cells which had been transfected with a specific siRNA and infected with JEV (MOI = 0.5) for 24 h.

**Additional file 9**. RT-PCR was conducted to measure the transcript levels of IFN β, ISG15, and Mx1 between METTL3 knockdown cells and control cells at 24 hpi and 36 hpi.

## Data Availability

The materials described in the manuscript will be made freely available to any scientist wishing to use them. All the data produced during this study have been included in the manuscript or its supplementary materials.
