# Supplementary material for: N6-methyladenosine modification positively regulate Japanese encephalitis virus replication
Source: Virol J. 2024 Jan 19;21:23. doi: 10.1186/s12985-023-02275-w (PMC10799421; doi:10.1186/s12985-023-02275-w)
Supplement: Supplementary file 2 — Additional file 2. A comprehensive overview of the MeRIP-seq data processing and alignment, MeRIP-seq peak-calling, motif discovery and metagene analysis is provided. [file 12985_2023_2275_MOESM2_ESM.pdf]

1 Library Construction and Sequencing

After total RNA was extracted, the enriched RNA was fragmented into short fragments (about 100nt) by using fragmentation buffer. RNA was divided into two parts, one of which was used as the input (no IP experiment was performed). The other RNA was enriched with m6A specific antibody. The enriched RNA was reverse transcripted into cDNA with random primers. Next, the cDNA fragments was end repaired, and ligated to Illumina sequencing adapters. Finally we got qualified library for sequencing, sequenced using Illumina NovaSeq^{TM} 6000 (or other platforms) by Gene Denovo Biotechnology Co. (Guangzhou, China)

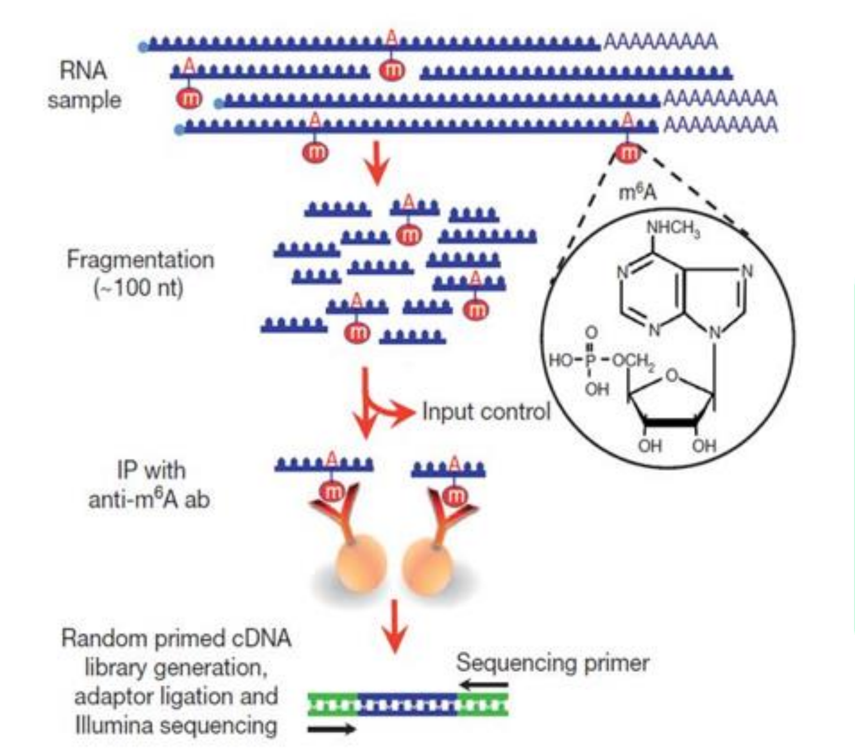

Fig 1 Flow chart of MeRIP-seq Library Construction

2 Flow Chart of Bioinformatics Analysis

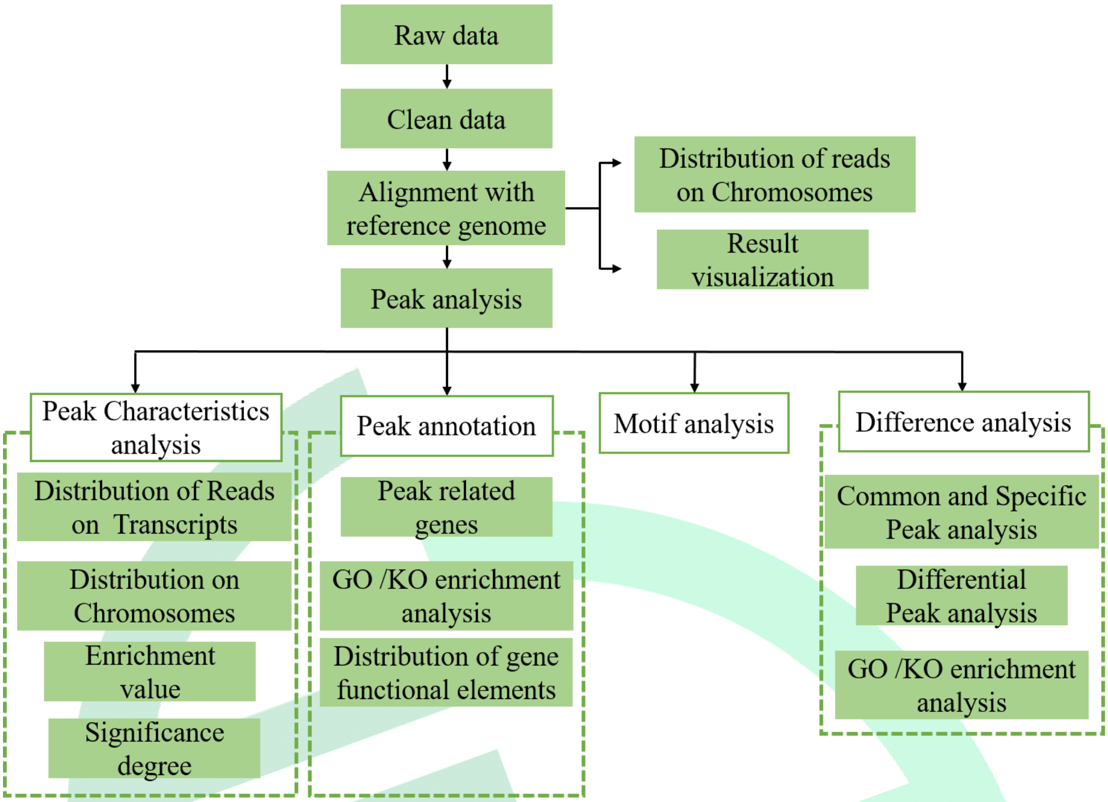

Fig 2 Flow chart of MeRIP-seq bioinformatic analysis

2.1 Clean Reads Filtering

Reads obtained from the sequencing machines included raw reads containing adapters or low quality bases which would affect the following assembly and analysis. Raw reads would be processed to get high quality clean reads according to four stringent filtering standards use fastp(version：0.20.0)[1] software:

i Removing reads containing adapters;

ii Removing reads containing more than 10% of unknown nucleotides (N);

iii Removing reads that are all A bases；

iv Removing low quality reads containing more than 50% of low quality (Q-value≤20) bases.

2.2 Alignment with reference genome

Short reads alignment tool Bowtie2 (2.2.8) [2] was used for mapping reads to ribosome RNA (rRNA) database. The rRNA mapped reads were then removed. The rRNA removed reads of each sample were then mapped to reference genome use HISAT2（version: 2.1.0）[3], respectively.

2.3 Peak Calling

exomePeak2 [4] (version: 1.0.0) software was used to identify read-enriched regions from MeRIP-seq data. Dynamic Poisson Distribution was used to calculated p-value of the specific region based on the unique mapped reads. The region would be defined as a peak when p-value<0.05.

Peak between duplicate samples in the group was filtered, and peaks with overlap more than 50% was retained for subsequent analysis. If there were two biological duplicates in the group, the retained peak must occur in both samples .If there were three biological

duplicates in the group, the retained peak must be present in at least two samples.

2.4 Peak related gene analysis

According to the genomic location information and gene annotation information of peak, peak related genes can be confirmed. Besides, the distribution of peak on different function regions, such as 5'UTR, CDS and 3'UTR, was performed.

2.5 Peak Related Genes GO Enrichment Analysis

Gene Ontology (GO) is an international standardized gene functional classification system which offers a dynamic-updated controlled vocabulary and a strictly defined concept to comprehensively describe properties of genes and their products in any organism. GO has three ontologies: molecular function, cellular component and biological process. The basic unit of GO is GO-term. Each GOterm belongs to a type of ontology. GO enrichment analysis provides all GO terms that significantly enriched in peak related genes comparing to the genome background, and filter the peak related genes that correspond to biological functions. Firstly all peak related genes were mapped to GO terms in the Gene Ontology database (http://www.geneontology.org/), gene numbers were calculated for every term, significantly enriched GO terms in peak related genes comparing to the genome background were defined by hypergeometric test. The calculating formula of P-value is:

$$P = 1 - \sum_{i=0}^{m-1} \frac{\binom{M}{i}\binom{N-M}{n-i}}{\binom{N}{n}}$$

Here N is the number of all genes with GO annotation; n is the number of peak related genes in N; M is the number of all genes that are annotated to the certain GO terms; m is the number of peak related genes in M. The calculated p-value were gone through Bonferroni Correction, taking corrected-pvalue≤0.05 as a threshold. GO terms meeting this condition were defined as significantly enriched GO terms in peak related genes. This analysis was able to recognize the main biological that peak related genes exercise.

2.9 Peak Related Genes Pathway Enrichment Analysis

Genes usually interact with each other to play roles in certain biological functions. Pathway- based analysis helps to further understand genes biological functions. KEGG is the major public pathway-related database (Release 87.0). Pathway enrichment analysis identified significantly enriched metabolic pathways or signal transduction pathways in peak related genes comparing with the whole genome background. The calculating formula is the same as that in GO analysis.

$$P = 1 - \sum_{i=0}^{m-1} \frac{\binom{M}{i}\binom{N-M}{n-i}}{\binom{N}{n}}$$

Here N is the number of all transcripts that with KEGG annotation, n is the number of peak related genes in N, M is the number of all transcripts annotated to specific pathways, and m is number of peak related genes in M. The calculated p-value was gone through FDR Correction, taking FDR ≤ 0.05 as a threshold. Pathways meeting this condition were defined as significantly enriched pathways in peak related genes.

2.6 Motif analysis

The interaction between protein and RNA were not random, while they showed some specific sequence preference. MEME suit (http://meme-suite.org/) and DREME (http://memesuite.org/tools/dreme) were used to detect the significant sequence motif in the transcript sequence associated with peaks.

2.7 Peak Combination and Multi-sample Clustering

The exomePeak2 [4] (version: 1.0.0) software was used to combine peaks of every group, and obtained the union of peaks among groups. Peak abundance was shown by calculating rpm-values of each sample. To compared samples, the unsupervised dimensionality reduction method principal component analysis (PCA) was applied in all samples using R package models (http://www.r-project.org/). PCA is a statistical procedure that converts thousands of correlated variables into a set of values of linearly uncorrelated variables called principal components.

2.8 Common and Specific Peak Related Genes Analysis between Groups

In different group, peaks with overlap were defined as the common peak. The genes closest to each peak were defined as peak-related genes. Peaks distant from peak-related gene (which locates farther upstream than 2k or downstream 300bp) were removed, and the remaining peak related genes were used for subsequent GO and KO enrichment analysis.

2.9 Differential Analysis of RNA Methylation Rate between Groups

The DiffBind [6] (version 2.8) software was used to analysis RNA methylation rate between groups. The relative methylation rate of each peak was calculated using MeRIP data and Input data. We identified peaks with $\log2|FC| \geq 1$ and FDR≤ 0.05 in a comparison as significant differential peaks for subsequent peak-related genes GO and KEGG enrichment analysis.

3 Reference

[1] Chen S, Zhou Y, Chen Y, et al. fastp: an ultra-fast all-in-one FASTQ preprocessor[J]. bioRxiv, 2018: 274100. (https://github.com/OpenGene/fastp)

[2] Langmead B, Salzberg S L. Fast gapped-read alignment with Bowtie 2[J]. Nature methods, 2012, 9(4): 357

[3] Kim, Daehwan, Ben Langmead, and Steven L. Salzberg. "HISAT: a fast spliced aligner with low memory requirements." Nature methods 12.4 (2015): 357.

[4] Jia Meng, Zhiliang Lu, A protocol for RNA methylation differential analysis with MeRIP-Seq data and exomePeak R/Bioconductor package, Methods. 2014, doi: 10.1016/j.ymeth.2014.06.008

[5] MEME suit : http://meme-suite.org/

[6] Stark R, Brown G. DiffBind: differential binding analysis of ChIP-Seq peak data[J]. R package version, 2011, 100: 4.3.
