# Supplementary figures and images for: N6-methyladenosine modification positively regulate Japanese encephalitis virus replication
Source: Virol J. 2024 Jan 19;21:23. doi: 10.1186/s12985-023-02275-w (PMC10799421; doi:10.1186/s12985-023-02275-w)

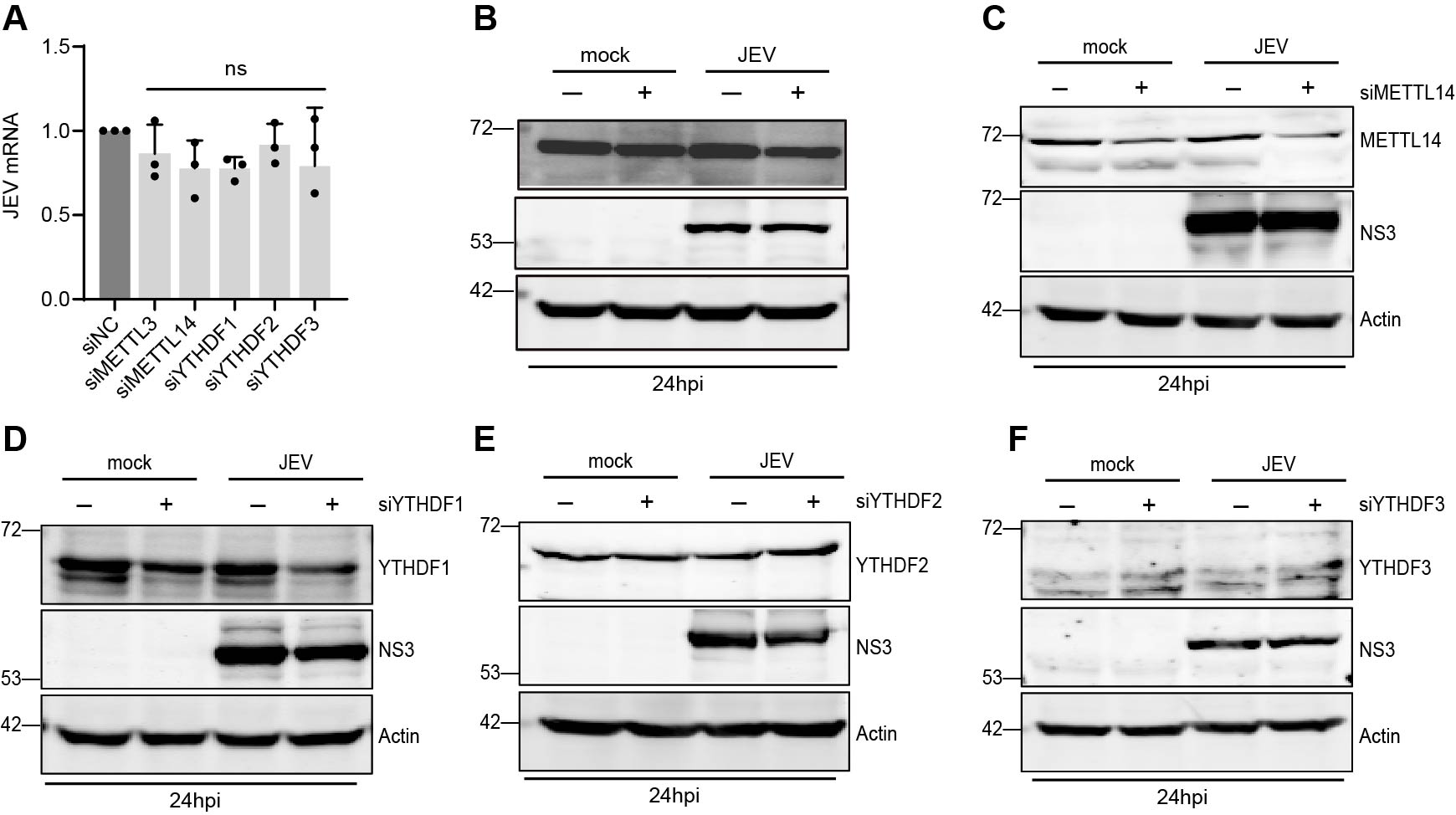

Supplement: Supplementary file 8 — Additional file 8. RT-PCR and Immunoblot analysis was conducted on neuro2a cells which had been transfected with a specific siRNA and infected with JEV (MOI = 0.5) for 24 h. [file 12985_2023_2275_MOESM8_ESM.jpg]

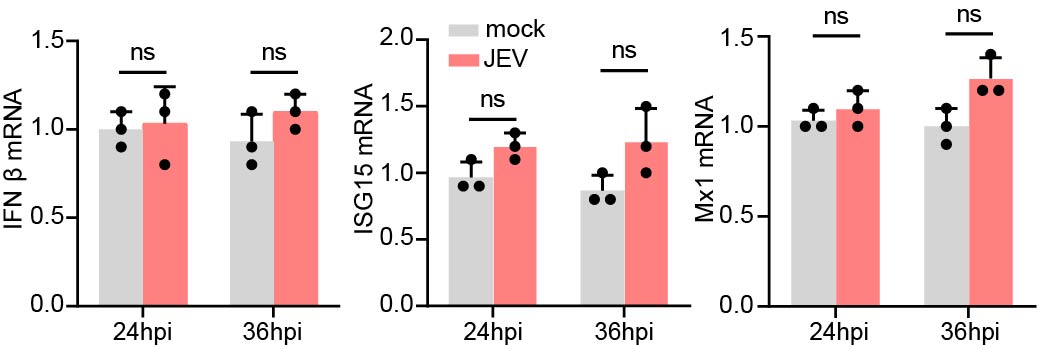

Supplement: Supplementary file 9 — Additional file 9. RT-PCR was conducted to measure the transcript levels of IFN β, ISG15, and Mx1 between METTL3 knockdown cells and control cells at 24 hpi and 36 hpi. [file 12985_2023_2275_MOESM9_ESM.jpg]
